# A comparison of breast cancer survival among young, middle-aged, and elderly patients in southern Iran using Cox and empirical Bayesian additive hazard models

**DOI:** 10.4178/epih.e2017043

**Published:** 2017-10-16

**Authors:** Samane Nematolahi, Seyyed Mohammad Taghi Ayatollahi

**Affiliations:** Department of Biostatistics, School of Medicine, Shiraz University of Medical Sciences, Shiraz, Iran

**Keywords:** Breast neoplasm, Prognosis, Additive hazards model, Bayesian inference, Cox regression analysis, Iran

## Abstract

**OBJECTIVES:**

A survival analysis of breast cancer patients in southern Iran according to age has yet to be conducted. This study aimed to quantify the factors contributing to a poor prognosis, using Cox and empirical Bayesian additive hazard (EBAH) models, among young (20-39 years), middle-aged (40-64 years), and elderly (≥ 65 years) women.

**METHODS:**

Data from 1,574 breast cancer patients diagnosed from 2002 to 2012 in the cancer registry of Fars Province (southern Iran) were stratified into 3 age groups. The Kaplan-Meier method was used to estimate the overall survival rates. Cox and EBAH models were applied to each age category, and the Akaike information criterion was used to assess the goodness-of-fit of the 2 hazard models.

**RESULTS:**

As of December 2012, 212 women (13.5%) in our study population had died, of whom 43 were young (15.3%), 134 middle-aged (11.8%), and 35 elderly (22.3%). The 5-year survival probability by age category was 0.83 (standard error [SE], 0.03), 0.88 (SE, 0.01), and 0.75 (SE, 0.04), respectively.

**CONCLUSIONS:**

The Nottingham Prognostic Index was the most effective prognostic factor. The model based on Bayesian methodology performed better with various sample sizes than the Cox model, which is the most widely used method of survival analysis.

## INTRODUCTION

Despite advances in cancer treatment, breast cancer is the second leading cause of mortality among women [1]. The most commonly diagnosed cancer in Western women is breast cancer, which affects approximately 1.7 million women annually worldwide [2].

Breast cancer is the most frequent type of cancer among Iranian women, and accounts for 24.6% of all cancers [3]. Research has shown that breast cancer affects Iranian women at least a decade sooner than their counterparts in developed countries, with the mean age ranging from 47.1 to 48.8 years [4].

The incidence of breast cancer has slowed down in developed countries, but in many developing countries, it has rapidly increased in the past few decades [5].

The World Health Organization reported that the incidence of breast cancer increased by more than 20% between 2008 and 2012, and that mortality increased by 14% within the same period [6]. The 5-year survival rate in Western countries has been estimated to be around 89% [6], while this rate has been reported to be 70, 89 and 58% in the Iranian cities of Shiraz, Tehran, and Semnan, respectively [5]. Recent studies have investigated prognostic factors associated with survival time. These factors include the stage of the disease, nodal status, tumor size, tumor grade, type of tumor, tumor histology, the number of involved nodes, the presence of lymphatic or vascular invasion, hormone receptor status, human epidermal growth factor receptor 2 status, the Nottingham Prognostic Index (NPI), and age [5,7-13]. The NPI is a numerical value that is computed by adding the values of tumor size (multiplied by a coefficient of 0.2), nuclear grade (1-3), and nodal status (1-3). The original NPI used a system that was divided into 3 categories, corresponding to good, moderate, and poor prognoses according to cut-off points of < 3.4, 3.4-5.4, and > 5.4, respectively [14].

Studies from various countries have shown that breast cancer is an aggressive disease that is especially difficult to control in young women, who have poorer survival than older patients. They are also at a higher risk of death than middle-aged women [7].

Annually, breast cancer incidence rates for women below the age of 40 and 40-49 years have increased since 1995 by 2.1 and 2.4%, respectively. The relative 10-year survival rate for women aged 50-69 years increased from 53% in 1975 to 75% in 2004 [11].

A study of outcomes according to age found that breast cancer in elderly patients was associated with an inferior prognosis in comparison to that of middle-aged women [15].

The majority of previous studies have analyzed survival data using the most widely used method, Cox regression analysis; they have not generally used Bayesian methodology due to its complex theoretical underpinnings. In addition, evaluating the covariates’ effects on absolute changes in the survival function has been a subject of interest for many researchers, which has led researchers to look for alternative models where an additive hazard regression model might be appropriate [16]. The objective of the present study was to determine potential prognostic factors in 3 age categories by using Cox and empirical Bayesian additive hazard (EBAH) models, and then to compare them using goodness-of-fit criteria such as the Akaike information criterion (AIC). The outcomes of our study will help health organizations to develop interventional programs for breast cancer patients.

## MATERIALS AND METHODS

### Study setting

According to the latest Iranian national census, Iran has a population of about 79,926,270 corresponding to 1.08% of the total world population [17]. Most studies relating to breast cancer have been conducted in the capital city, Tehran. The present study was conducted in Shiraz, the capital of Fars Province, which is located in the southwest of Iran and has a population of 4,851,274 people [17]. The data were collected from the Nemazi Hospital, the oldest cancer registry office in southern Iran, which was established in Shiraz in 1971 [18].

### Data source and subjects

Cancer patients were registered at the Nemazi Hospital. For our study, we collected data from January 2002 through December 2012. After excluding patients with other kinds of cancer and incomplete data, a total of 1,574 patients were included in this study. Patients’ medical records included skin involvement, estrogen receptor status, progesterone receptor status, the total number of affected lymph nodes , lymphatic involvement, vascular involvement, perineural involvement, resected margin, deep margin, stage of disease, age at diagnosis, tumor size, and nuclear grade. All patients had undergone chemotherapy.

We categorized breast cancer patients into young women (20-39 years), middle-aged women (40-64 years), and elderly women (65 years and older), then analyzed each group separately.

The survival time of each patient was determined as the difference in time (years) between the date of initial diagnosis until the date of expiry through breast cancer or the end of follow-up. The censoring mechanism in this study was generalized type I censoring; all patients had to be present until the end of the study, and no cases were lost to follow-up.

### Prognostic factors

We examined the effects of age at diagnosis; hormonal receptor status (estrogen and progesterone; positive or negative); skin, perineural, resected margin, deep margin, lymphatic, and vascular involvement (involved, or free of involvement); and the NPI on the survival time of breast cancer patients in 3 age groups. The age at diagnosis was a continuous variable. Stage at diagnosis was classified as stage I, stage II (including stage IIA and IIB), and stage III (including stage IIIA, IIIB, and IIIC). The tumor, node, metastasis staging system was used to divide the patients into stages, and no stage IV patients were present. Nodal status was classified into 3 categories depending on the number of nodes involved (0, 1-3, or > 3). Tumor size was classified as < 3, 3-5, or > 5 cm. The nuclear grade was classified into 3 stages: well differentiated, poorly differentiated, and undifferentiated. The NPI is a numerical value computed by adding the measure of tumor diameter (multiplied by a coefficient of 0.2), nodal status (1-3), and nuclear grade (1-3). The NPI is divided into 3 classifications, corresponding to good, moderate, and poor prognostic groups (< 3.4, 3.4-5.4, and > 5.4, respectively) [14]. Some variables, such as stage of diagnosis, nodal status, tumor size, and nuclear grade were not entered into the final model to avoid the problem of multicollinearity.

### Statistical analysis

The 5-year survival of the patients with breast cancer belonging to 3 age groups (young, middle-aged, and elderly women) was calculated by the Kaplan-Meier method. In this study, we used Cox and EBAH models to examine the prognostic factors influencing patients’ survival time.

The variables of age, skin involvement, estrogen receptor status, progesterone receptor status, lymphatic involvement, perineural involvement, resected margin involvement, deep margin involvement, vascular involvement, and the NPI were chosen to be entered into the final model using univariate Cox regression analysis. Disease stage had a strong correlation with nodal status, and as a result this variable was not used in the final model.

The proportional hazard assumptions of the Cox model were checked on the basis of Schoenfeld residuals and confirmed for all predictor variables. The regression parameters (β) in Cox models can be interpreted as the logarithm of the hazard ratio (HR) for every unit increment of the corresponding covariate *x*.

$$\mathrm{EBAH}\;\mathrm{model}:\;\;\;h(t|X)=\;h_{0}(t)\;+\;\beta_{1}X_{1}\;+\;\ldots \;+\;\beta_{p}X_{p}=\;h_{0}(t)\;+\;\beta ´X$$

$$\mathrm{Cox}\;\mathrm{regression}\;\mathrm{hazard}\;\mathrm{model}:\;\;h(t|X)=\;h_{0}(t)\;\exp(\beta_{1}X_{1}\;+\;\ldots \;+\;\beta_{p}X_{p})=h_{0}(t)\;\exp(\beta ´X)$$

In contrast to the Cox model, which estimates HRs, an additive hazard regression model estimates the differences in HR h(t | x1) – h(t | x2)= βT(x1 − x2) and the survival ratio (SR) S(t | x1)/S(t | x2)= exp{−tβT(x1 − x2)} for 2 subjects. Therefore, when the attribute risk or SR is of primary interest, or the proportional hazard assumption is violated, an additive hazard regression model may be more appropriate. Bayesian survival analysis consists of data and prior information, it generates conclusions based on the synthesis of new information from observed data and historical knowledge. Bayesian inference can be performed either with a full Bayesian or empirical Bayesian posterior analysis, Empirical Bayesian methods are procedures for statistical inference in which the prior distribution is estimated from the data, while in full Bayesian methods the prior distribution is fixed before any data are observed. Therefore, the full Bayesian method is appropriate when one has complete and precise prior information; otherwise, the empirical Bayesian method is advised. Model estimation and inference in the full Bayesian approach is based on Markov-chain Monte Carlo simulations. The empirical Bayesian method for parameter estimation in additive hazard regression models is based on the mixed model representation of penalized regression models with inferences based on penalized maximum likelihood and marginal likelihood estimation (a generalization of restricted maximum likelihood estimation). In fact, additive models can always be expressed as a generalized linear mixed model (GLMM). Based on the GLMM representation, regression and variance parameters can be estimated using iteratively weighted least squares and approximate marginal or restricted maximum likelihood techniques, which were developed for GLMMs [16].

A full discussion can be found in the methodology manual of the open-source statistical package Bayes X .

Kaplan-Meier and Cox regression analyses were performed using SPSS version 23 (IBM Corp., Armonk, NY, USA) and the EBAH model was fitted by Bayes X version 2.1 (German Research Foundation, Munich, Germany).

## RESULTS

As of December 2012, 212 women (13.5%) in our study population had died as a result of breast cancer, of whom 43 were aged 20-39 years (15.3%), 134 were aged 40-64 years (11.8%), and 35 were aged ≥ 65 years (22.3%).

The characteristics of the study subjects in the 3 age categories are presented in Table 1. The mean age of the patients at the time of diagnosis was 34.72, 50.36, and 72.10 years in the young, middle-aged, and elderly women, respectively. The most frequent age category was that of middle-aged patients, who comprised 72.2% of the sample. Most frequently, the women in all 3 age groups had stage II breast cancer as their primary diagnosis (44.5, 44.4, and 47.8% in young, middle-aged, and elderly patients). The mean tumor size was 3.03 cm in the young patients, which was greater than the other 2 age groups. The plurality of the patients in each age group had no lymph node involvement (45.6, 51.6, and 50.3% in young, middle-aged, and elderly patients, respectively), although in younger women the proportion of patients with > 3 involved nodes was larger than in the other 2 age groups. Most patients in each age group did not have skin, vascular, or perineural involvement, and the majority of patients in all 3 groups had hormonal receptor-positive tumors. The most frequent nuclear grade in the 3 age groups was poorly differentiated (52.0, 52.0, and 55.4% in young, middle-aged, and elderly patients, respectively). Most of the patients had lymphatic, resected margin, and deep margin involvement. The NPI was moderate in 48.0% of the young women, while 32.7 and 19.3% of patients had good and poor prognoses, respectively. In the middle-aged women, 49.2% had a moderate NPI, while 35.1 and 15.7% had good and poor prognoses, respectively. The NPI of 45.3% of the elderly patients was moderate, while 36.9 and 17.8% had good and poor prognoses, respectively.

The 5-year survival probability after the breast cancer diagnosis was estimated to be 0.83 (standard error [SE], 0.03), 0.88 (SE, 0.01) and 0.75 (SE, 0.04) in young, middle-aged, and elderly women, respectively.

**Figure 1.**
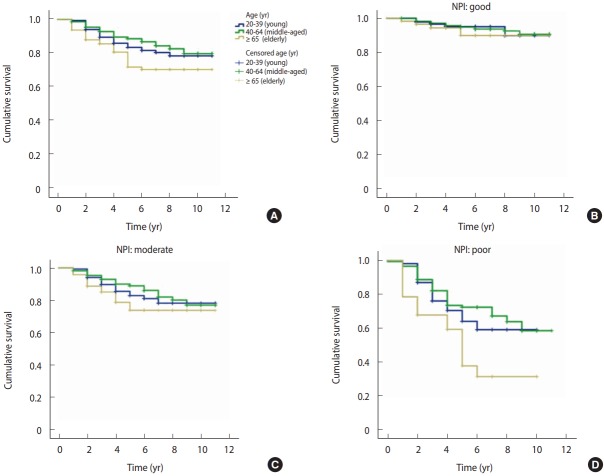
Kaplan-Meler survival curves of (A) breast cancer patients and in (B) good, (C) moderate, and (D) poor level of NPI in three age categories. NPI, Nottingham Prognostic Index.

The survival rate of young women was non-significantly different from that of the elderly group, but significantly lower than that of the middle-aged group (Figure 1A).

The NPI is one of the most important grading systems, and in this study, it was confirmed to be one of the most effective prognostic factors for predicting the survival of the breast cancer patients in the 3 different age groups. The survival of patients with a good NPI prognosis was similar in the 3 age groups, but the between-group differences increased in patients in all 3 groups with a moderate or poor NPI (Figure 1B-1D).

Cox regression analysis showed that age at the time of diagnosis (HR, 0.88; p= 0.001) and the NPI (HR, 5.26; p= 0.006) were significantly associated with survival time in young women, and the influential prognostic factors in middle-aged patients were skin involvement (HR, 1.99; p=0.03), perineural involvement (HR, 1.79; p= 0.005), progesterone receptor negativity (HR, 0.61; p= 0.03), age at the time of diagnosis (HR, 1.05; p= 0.001), and the NPI (HR, 5.01; p< 0.001). Of these factors, only the NPI (HR, 9.63; p= 0.002) had a significant effect on the survival time of elderly patients (Table 2).

In the young women, the EBAH analysis showed a significant effect of age (SR, 1.14; p< 0.001) and NPI (SR, 0.19; p= 0.006) on the survival time. The significant prognostic factors affecting survival time in middle-aged patients were age at diagnosis (SR, 0.95; p < 0.001), skin involvement (SR, 0.51; p=0.03), perineural involvement (SR, 0.56; p= 0.005), progesterone receptor negativity (SR, 1.64; p= 0.03), and the NPI (SR, 0.43; p= 0.002 and SR, 0.19; p< 0.001). The survival time of the elderly patients was affected only by the NPI (SR, 0.10; p = 0.002). Of note, the following factors showed no significant associations with the risk of mortality: resected margin involvement, deep margin involvement, lymphatic involvement, vascular involvement, and estrogen receptor positivity (p> 0.05 for all). The estimates of the HR and SR associated with significant prognostic factors in both hazard models indicate that the risk of mortality decreased with an increased age at diagnosis in young patients, but increased in middle-aged patients. In other words, the effect of age at diagnosis in young and middle-aged patients was opposite. The risk of death in middle-aged patients with skin involvement was significantly greater than in those without skin involvement. The survival rate in patients with perineural involvement and progesterone receptor negativity was lower. NPI affected patients’ survival rate in all 3 age groups, but it had the strongest effect in the elderly group (Table 2).

When comparing the results of the 2 hazard models, we observed that the Cox regression analysis and EBAH models showed similar significant prognostic factors, so we computed the AIC as a goodness-of-fit criterion and compared them. The AIC values of the EBAH model were lower than those of the Cox regression analysis model, so we concluded that the model based on Bayesian methodology was more suitable for survival analysis with different sample sizes (Table 2).

In addition, a comparison of the SR and HR formulas showed that there were differences in the positive and negative signs in the exponential term, which is why the directions of the SR and HR were different.

## DISCUSSION

Globally, the surge in breast cancer incidence and mortality represents a significant and growing threat for developing countries. In this study, our aim was to compare the Cox proportional hazard and EBAH models for survival data related to 1,574 women diagnosed with breast cancer divided into 3 groups of young, middle-aged, and elderly patients. These 2 models consider different relationships between the hazard and covariates. No previous studies have analyzed survival data using an EBAH model. In this paper, the covariates that were selected to remain in the final model were similar in these 2 models for the 3 age categories. The signs of the estimated coefficients in the 2 models were also similar, but their coefficients were different in magnitude. This is not surprising, because the coefficients of Cox models are related to risk ratios, while those of EBAH models are related to risk differences [19].

In Western countries, the majority of breast cancer patients are women aged between 60 and 70, but in this study, most patients were middle-aged. Previous studies have shown that young women had poorer survival than other age groups [20]. In our study, elderly patients had a lower chance of survival than the other 2 age groups, but the percentage of women with breast cancer in this group was lower.

Poor survival can be explained by the absence of or inadequate access to early diagnosis and treatment.

Our study demonstrated that the NPI was the most effective prognostic factor in our study; this prognostic factor was the only one predictive of survival in elderly patients. Our findings concur with those of several studies carried out in different parts of the world [8,13,21,22].

In the present study, the 5-year overall survival rate of young patients was 83.0%,which is higher than was reported in Uganda (56.0%) in 2008, China (76.5%), and Taiwan (78.4%). In the middle-aged patients, this rate has been estimated to be 88.0%, which is lower than has been reported in Sweden (89.0%) and equal to what has been reported in the US (88.0%). In elderly patients, this rate was 75.0%, which is less than has been reported in South Korea (83.5%), North Vietnam (85.1%), Sweden (89.0%), Canada (86.0%), and the US (88.0%). These differences are attributable to variations in lifestyle and environmental exposure. Progesterone receptor positivity was an important prognostic factor for survival in middle-aged women with breast cancer in this study. Recent studies have shown that testing for progesterone receptor status helped doctors to choose the best treatment method and reduced breast cancer mortality [23,24].

In consistent associations between the survival rate and skin involvement have been reported in studies conducted around the world [10,13,21]. Our study found a negative effect of skin involvement status on the survival of middle-aged women, but no effect on the survival rates of young or elderly patients.

Another important prognostic factor in young and middle-aged patients was age at diagnosis. In young patients, those who were younger at diagnosis showed a poorer prognosis, but increased age increased the risk of death in middle-aged patients. This result in middle-aged women is consistent with the results of previous studies [10,13,21]. Perineural invasion is the process of neoplastic invasion of the nerves; it has been rarely observed in mammary carcinoma, and therefore has not been studied extensively. In the present study, it was not a significant prognostic factor in young and elderly breast cancer patients, but perineural involvement was associated with poor survival in the middle-aged patients. A previous study showed that perineural invasion did not affect survival outcomes [25], while another study concluded that it did affect breast cancer patients’ survival rate [26].

Some strengths of our study include there porting of HRs and SRs calculated using 2 different survival models. We compared 2 survival models with different sample sizes in order to provide information helpful for the diagnosis and treatment of patients in different age categories.

## Figures and Tables

**Table 1.** Characteristics of young, middle-aged, and elderly breast cancer patients in southern Iran

Characteristics	Young (n=281)	Middle-aged (n=1,136)	Elderly (n=157)
Age at diagnosis (yr)	34.72±3.80	50.36±6.53	72.10±6.04
Stage			
I	60 (21.4)	296 (26.1)	35 (22.3)
II	125 (44.5)	504 (44.4)	75 (47.8)
III	96 (34.2)	336 (29.6)	47 (29.9)
Tumor size (cm)			
Mean±SD	3.03±1.71	2.94±1.69	3.02±1.58
Tumor size group (cm)			
<3	202 (71.9)	855 (75.3)	114 (72.6)
3-5	61 (21.7)	223 (19.6)	35 (22.3)
>5	18 (6.4)	58 (5.1)	8 (5.1)
Nodal status			
0	128 (45.6)	586 (51.6)	79 (50.3)
1-3	75 (26.7)	262 (23.1)	40 (25.5)
>3	78 (27.8)	288 (25.3)	38 (24.2)
Nuclear grade			
Well differentiated	94 (33.5)	381 (33.5)	49 (31.2)
Poorly differentiated	146 (52.0)	591 (52.0)	87 (55.4)
Undifferentiated	41 (14.5)	164 (14.5)	21 (13.4)
Skin involvement			
Involved	17 (6.0)	47 (4.0)	11 (7.0)
Free	264 (94.0)	1,089 (95.9)	146 (93.0)
Lymphatic involvement			
Involved	146 (52.1)	578 (51.2)	81 (51.6)
Free	134 (47.9)	551 (48.8)	76 (48.4)
Vascular involvement			
Involved	49 (17.5)	233 (20.6)	39 (25.0)
Free	231 (82.5)	900 (79.4)	117 (75.0)
Perineural involvement			
Involved	35 (12.5)	200 (17.7)	31 (19.7)
Free	244 (87.5)	929 (82.3)	126 (80.3)
Resected margin involvement			
Involved	251 (89.6)	1,059 (93.5)	144 (91.7)
Free	29 (10.4)	74 (6.5)	13 (8.3)
Deep margin			
Involved	253 (90.7)	1,053 (93.0)	146 (93.0)
Free	26 (9.3)	79 (7.0)	11 (7.0)
Estrogen receptor			
Positive	183 (66.5)	791 (71.0)	113 (74.3)
Negative	92 (33.5)	323 (29.0)	39 (25.7)
Progesterone receptor			
Positive	153 (56.2)	683 (61.7)	95 (62.9)
Negative	119 (43.8)	424 (38.3)	56 (37.1)
NPI			
Good	92 (32.7)	399 (35.1)	58 (36.9)
Moderate	135 (48.0)	558 (49.2)	71 (45.3)
Poor	54 (19.3)	178 (15.7)	28 (17.8)

Values are presented as number (%) or mean±standard deviation. NPI, Nottingham Prognostic Index.

**Table 2.** Results of Cox regression analysis and the empirical Bayesian additive model in 3 age categories, including prognostic factors

Variables	Young		Middle-aged		Elderly	
	SR	HR	SR	HR	SR	HR
Intercept	2.17		803.08^***^		756.50^**^	
Resected margin involvement (involved vs. free)	3.00	0.35	1.68	0.59	0.92	1.04
Skin involvement (involved vs. free)	0.45	2.28	0.51^*^	1.99^*^	0.79	1.17
Deep margin (involved vs. free)	1.21	0.79	0.89	1.11	1.07	0.97
Lymphatic involvement (involved vs. free)	0.55	1.81	0.97	1.04	1.85	0.55
Vascular involvement (involved vs. free)	0.85	1.14	0.86	1.16	0.69	1.44
Perineural involvement (involved vs. free)	0.84	1.18	0.56^**^	1.79^**^	0.85	1.15
Estrogen receptor (negative vs. positive)	2.23	2.18	0.82	1.22	1.23	0.79
Progesterone receptor (negative vs. positive)	0.60	0.60	1.64^*^	0.61^*^	0.53	0.55
Age at diagnosis	1.14^***^	0.88^***^	0.95^***^	1.05^***^	0.98	1.02
NPI 1 (moderate vs. good)	0.42	2.35	0.43^**^	2.32^**^	0.32	3.15
NPI 2 (poor vs. good)	0.19^**^	5.26^**^	0.19^***^	5.01^***^	0.10^**^	9.63^**^
AIC	319.36	387.37	1,038.15	1,440.79	246.46	278.25

SR, survival ratio; HR, hazard ratio; NPI, Nottingham Prognostic Index; AIC, Akaike information criterion.

* p<0.05,

** p<0.01,

*** p<0.001.
